# Fermented Pangasius Catfish Waste Improves Energy Utilization and Nutrient Digestibility in Low‐Protein Diets for Indonesian Super Native Chickens

**DOI:** 10.1002/vms3.71126

**Published:** 2026-08-05

**Authors:** Abun Abun, Kiki Haetami, Rahmad Fani Ramdhan

**Affiliations:** ^1^ Department of Animal Nutrition and Feed Technology, Faculty of Animal Husbandry Universitas Padjadjaran Sumedang West Java Indonesia; ^2^ Department of Fisheries, Faculty of Fisheries and Marine Science Universitas Padjadjaran Sumedang West Java Indonesia

**Keywords:** digestibility, fermented fish by‐products, indigenous chickens, metabolizable energy, protein‐reduced diets

## Abstract

**Background:**

Low‐protein poultry diets require locally available ingredients that preserve nutrient use and digestive function under practical production conditions. Fermented fish‐processing by‐products may improve feeding value, but evidence for Indonesian super native chickens remains limited.

**Objectives:**

To evaluate fermented pangasius catfish waste (FPCW) as a local feed ingredient in low‐protein diets for Indonesian super native chickens and to identify an inclusion level that optimizes energy utilization and nutrient digestibility.

**Methods:**

A total of 240‐day‐old chickens were reared on a starter diet containing 18% crude protein until 5 weeks of age. From Weeks 6 to 10, birds were assigned to six dietary treatments in a completely randomized design with four replicates of 10 birds each. The treatments comprised a low‐protein control (15% crude protein), the same basal diet supplemented with 5%, 10%, 15% or 20% FPCW, and a higher protein control (18% crude protein). Samples collected at 11 weeks of age were used to determine apparent metabolizable energy (AME), nitrogen‐corrected AME (AMEn), nitrogen retention, apparent dry matter and crude‐protein digestibility, selected ileal bacterial counts and digestive enzyme activities.

**Results:**

Diet significantly affected AME, AMEn, nitrogen retention, apparent dry matter digestibility, apparent crude‐protein digestibility, protease activity, lipase activity and ileal *Staphylococcus aureus* counts (*p* < 0.05). The 10% FPCW diet produced the highest AME (2948 kcal/kg) and AMEn (2792 kcal/kg) and improved apparent dry matter and crude‐protein digestibility to 63.76% and 72.90%, respectively. Nitrogen retention in the 10% FPCW group was comparable with that in the higher protein control. Total viable bacteria and *Escherichia coli* were not affected by diet, whereas *S. aureus* counts increased at higher inclusion levels. Lipase activity was greatest at 5%–10% FPCW inclusion.

**Conclusions:**

FPCW can be incorporated into low‐protein diets for Indonesian super native chickens, with 10% inclusion providing the most favourable balance between energy utilization and nutrient digestibility. The response to higher inclusion levels was less favourable, indicating that processing benefits must still be balanced against dietary matrix constraints.

## Graphical abstract

Fermented pangasius catfish waste improved nutrient use in low‐protein diets for Indonesian super native chickens. A 10% inclusion level produced the highest apparent metabolizable energy, nitrogen‐corrected AME, and dry matter and crude‐protein digestibility, whereas higher inclusion levels were less favourable. The ingredient, therefore, shows value as a local nutrient‐utilization enhancer, provided its dietary inclusion level is carefully controlled.

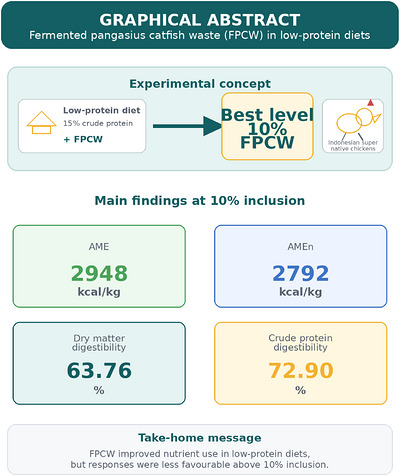

## 1 Introduction

Fish‐processing by‐products are important alternative nutrient sources for non‐ruminant feeding systems. However, direct use is limited by high moisture content, rapid spoilage and variable nutritional quality (Brito et al. 2020; Dadkhodazadeh et al. 2024). Protein‐ and lipid‐rich fractions are particularly vulnerable to proteolysis, oxidation and microbial deterioration during handling and storage (Abun et al. 2025a). In Indonesia, pangasius processing generates residual biomass that can be redirected into value‐added feed ingredients rather than discarded as waste (Ramadhan et al. 2016).

Fermentation is a biological upgrading strategy for fish by‐products and other unconventional feed resources. Depending on substrate composition and inoculum, fermentation can partially hydrolyse proteins and lipids, reduce undesirable compounds and increase soluble nutrients and small peptides (Dadkhodazadeh et al. 2024; Li and Wang 2021; Sukhikh et al. 2022). Lactic acid bacteria, *Bacillus* spp. and yeast‐based systems can contribute proteolytic activity, microbial stabilization and changes in nutrient availability during feed bioconversion (Heidari et al. 2024; Lim et al. 2019; Contesini et al. 2018; Ye et al. 2021). These effects are relevant to veterinary production because ingredient quality influences digestive function, nutrient utilization, gut ecology and the resilience of birds reared under practical farm conditions (Elbaz et al. 2023; Beckman et al. 2024).

In poultry nutrition, these advantages are particularly relevant when formulating low‐protein diets. Protein‐reduced diets can reduce feed cost and nitrogen output, but they must maintain metabolizable energy supply, amino acid balance, nutrient digestibility and gastrointestinal function to avoid performance losses (Dastar et al. 2025; Shabani et al. 2021). Tropical broiler production is also constrained by heat stress, variable feed quality and management limitations (Kpomasse et al. 2021). In recent nutritional studies, methionine supplementation helped support production efficiency and heat resilience in low‐protein diets (Songuine et al. 2025), nutritional plane and organic acid supplementation altered broiler responses (Ogunola et al. 2025), and dietary fibre, enzyme supplementation and processed feather meal affected ileal digestibility, intestinal morphology or performance outcomes (Pourazadi et al. 2024; Salehizadeh et al. 2025). These findings indicate that the success of protein‐reduced feeding strategies depends on ingredient processing, inclusion level and bird genotype rather than on crude‐protein reduction alone (Elbaz et al. 2023; Beckman et al. 2024; Dastar et al. 2025; Shabani et al. 2021; Abun et al. 2022; Mirzah et al. 2020).

Information on fermented pangasius catfish waste (FPCW) in Indonesian super native chickens remains limited. Previous work described the nutritional value and physical properties of fermented pangasius waste (Abun et al. 2025a; Abun et al. 2025b). However, a treatment‐level comparison of energy utilization, nutrient digestibility, digestive enzyme activity and selected ileal bacterial indicators in low‐protein diets for this chicken type has not been clearly presented. Indonesian super native chickens are relevant to tropical production systems because they are widely used as local meat‐type birds and are commonly raised under resource‐sensitive feeding conditions (Kpomasse et al. 2021).

Therefore, this study evaluated graded inclusion levels of FPCW in low‐protein diets for Indonesian super native chickens. We hypothesized that a moderate inclusion level would improve apparent metabolizable energy (AME), nitrogen‐corrected AME (AMEn) and nutrient digestibility while supporting digestive enzyme activity but would not necessarily improve all selected bacterial endpoints.

## 2 Materials and Methods

### 2.1 Ethics Statement

Animal care and sampling procedures were conducted in accordance with institutional requirements, and efforts were made to minimize animal suffering and the number of birds used.

### 2.2 Study Design and Analytical Framework

The feeding trial used a single dietary design, whereas endpoint measurements were generated in separate analytical modules after the same treatment period. Accordingly, energy utilization, digestibility, microbiology and enzyme activity data were analysed and interpreted at the treatment level, with the replicate serving as the experimental unit across modules.

### 2.3 Preparation of FPCW

Pangasius catfish waste was washed, chopped, homogenized and steamed at 90°C for 15 min to reduce the initial microbial load. Pure cultures of *Lactobacillus paracasei*, *Bacillus subtilis* and *Saccharomyces cerevisiae* were separately adapted to the substrate by fermenting 100 g of steamed fish waste with 1.5 L sterile water and 10% starter culture in closed containers for 5 days at room temperature. Fermentation and post‐processing followed a controlled bioconversion approach similar to that used for other aquatic by‐products and fermented feed substrates (Dadkhodazadeh et al. 2024; Li and Wang 2021; Heidari et al. 2024). The substrate and microbial consortium were specific to the present pangasius waste system (Abun et al. 2025b). The chemical composition reported in Table 1 was determined by an accredited commercial laboratory in Indonesia; the certificate of analysis is available from the corresponding author on reasonable request.

**TABLE 1:** Chemical composition of pangasius catfish waste before and after fermentation.

Nutrient	Before fermentation	After fermentation
Crude protein (%)	26.05	37.27
Ether extract (%)	20.94	10.51
Crude fibre (%)	2.21	1.15
Calcium (%)	1.50	5.56
Phosphorus (%)	7.20	8.60
Lysine (%)	1.29	1.95
Methionine (%)	0.40	0.50
Cystine (%)	0.26	0.59
Total essential amino acids (%)	9.43	16.89
Total non‐essential amino acids (%)	14.21	17.62
Total amino acids (%)	23.65	34.50
Omega‐3 (%)	0.22	0.50
Omega‐6 (%)	0.72	1.82
Omega‐9 (%)	0.30	0.76
Unsaturated fatty acids (%)	5.87	12.68
Saturated fatty acids (%)	8.25	5.75
Total fatty acids (%)	14.12	18.43

*Note*: Values are percentages unless otherwise indicated.

*Source*: Accredited commercial laboratory analysis (2023).

### 2.4 Experimental Diets

Experimental diets were prepared as pellets for the finisher phase of Indonesian super native chickens. Two control diets were formulated: a low‐protein control containing 15% crude protein (R0) and a higher protein control containing 18% crude protein (RH). Both control diets were formulated to provide approximately 2750 kcal metabolizable energy/kg. The treatment diets consisted of the low‐protein basal formula supplemented with 5%, 10%, 15% or 20% FPCW (R1–R4). Ingredient composition and calculated nutrient values are presented in Table 2.

**TABLE 2:** Ingredient composition and calculated nutrient content of the experimental diets.

Ingredient/Nutrient	R0	R1	R2	R3	R4	RH
Fish meal (%)	10.00	8.00	4.50	2.00	0.00	13.00
FPCW (%)	0.00	5.00	10.00	15.00	20.00	0.00
Soybean meal (%)	8.00	6.00	5.00	3.50	1.50	14.00
Yellow corn (%)	57.00	58.00	58.00	58.00	58.00	53.00
Rice bran (%)	23.00	21.00	20.50	19.50	18.00	18.00
CaCO_3_ (%)	0.75	0.75	1.00	1.50	2.00	0.75
Bone meal (%)	0.75	0.75	0.50	0.00	0.00	0.75
Premix (%)	0.50	0.50	0.50	0.50	0.50	0.50
Crude protein (%)	15.07	15.09	15.00	15.06	15.09	18.07
Ether extract (%)	5.19	5.27	5.39	5.50	5.59	5.61
Crude fibre (%)	4.20	3.92	3.83	3.67	3.43	3.96
Calcium (%)	1.09	1.25	1.37	1.59	1.95	1.26
Phosphorus (%)	0.53	0.90	1.18	1.45	1.82	0.62
Lysine (%)	1.10	1.04	0.94	0.88	0.81	1.37
Methionine (%)	0.49	0.47	0.44	0.42	0.41	0.54
Cystine (%)	0.30	0.29	0.29	0.29	0.29	0.33
ME (kcal/kg)	2750	2763	2748	2742	2746	2751

*Note*: R0, low‐protein control diet without fermented pangasius catfish waste; R1–R4, diets containing 5%, 10%, 15% or 20% fermented pangasius catfish waste; RH, higher protein control diet without fermented pangasius catfish waste.

Abbreviation: FPCW, fermented pangasius catfish waste.

### 2.5 Birds, Management and Experimental Design

A total of 240‐day‐old Indonesian super native chickens were used. From hatch to 35 days of age, all birds received a starter diet containing 18% crude protein. At 6 weeks of age, birds were allocated to six dietary treatments in a completely randomized design with four replicates per treatment and 10 birds per replicate. The treatment period lasted 35 days, from Weeks 6 to 10, and endpoint sampling was conducted when the birds were 11 weeks old. Birds were housed by replicate under routine management conditions, and feed and water were provided according to the experimental programme.

### 2.6 Sample Collection and Analytical Locations

The feeding trial, bird management, feed preparation and endpoint sampling were conducted at the Laboratory of Poultry and Non‐Ruminant Nutrition and the Mini Feed mill, Faculty of Animal Husbandry, Universitas Padjadjaran, Sumedang, West Java, Indonesia. Feed, excreta, digesta and digestive‐tract samples were processed for proximate‐, energy‐, microbiological‐ and enzyme‐related analyses at the Feed Research and Testing Laboratory, Faculty of Animal Husbandry, Universitas Padjadjaran, Sumedang, West Java, Indonesia. Chemical characterization of FPCW for Table 1 was performed by an accredited commercial laboratory in Indonesia, with the laboratory certificate retained by the corresponding author.

For the energy utilization module, birds sampled from each replicate were transferred to labelled metabolic cages after the treatment period. Birds were fasted for 24 h to empty the digestive tract, then offered 120 g of the corresponding treatment diet. Total excreta were collected during the following 24 h according to a total‐collection approach used for poultry energy evaluation (Kong and Adeola 2014; Mahmoud and Ravindran 2025). Excreta were sprayed every 3 h with 5% borax solution to minimize nitrogen loss, cleaned of feathers, weighed and stored pending analysis.

For the digestibility module, birds were slaughtered at the end of the treatment period, and hindgut digesta/faeces were collected from the large intestine. Samples were stored in labelled containers and analysed for dry matter, crude protein and lignin. Lignin in feed and faeces was used as an internal marker for apparent digestibility calculations (van Soest 1979).

### 2.7 Chemical, Microbial and Enzyme Analyses

Dry matter and crude protein in feed, excreta and faecal samples were determined using standard proximate‐analysis procedures, including oven drying for dry matter and Kjeldahl nitrogen determination for crude protein (AOAC International 2019). Gross energy of feed and excreta was determined by bomb calorimetry. Lignin was determined according to the marker approach described by van Soest (1979). The analytical workflow followed the principle that energy and nutrient utilization estimates for poultry feed ingredients should be based on defined sampling, laboratory and calculation procedures (Kong and Adeola 2014; Mahmoud and Ravindran 2025).

Ileal digesta for microbiological analysis were collected aseptically, serially diluted in sterile physiological saline and enumerated by plate‐count procedures. Total viable bacteria were determined by the total plate‐count method. *Escherichia coli* was enumerated on Eosin Methylene Blue agar, and *Staphylococcus aureus* was enumerated on mannitol salt agar after incubation at 37°C for 24 h. Results were expressed as colony‐forming units per gram of sample according to the scale shown in Table 5.

Duodenal samples were collected for protease and lipase assays. Protease activity was determined calorimetrically using a tyrosine standard curve in *Tris*‐HCl buffer. Lipase activity was determined titrimetrically using palm oil as the substrate. Protease activity was expressed as U/mL, where 1 U was defined as the amount of enzyme releasing 1 µmol tyrosine/min under the assay conditions. Lipase activity was expressed as U/mL, where 1 U was defined as 1 µmol fatty acid released/min.

### 2.8 Calculations

The main response variables were AME, AMEn, nitrogen retention, apparent dry matter digestibility, apparent crude‐protein digestibility, selected ileal bacterial counts and digestive enzyme activities. AME and AMEn were calculated using gross energy intake, excreta energy output, feed intake and nitrogen retention following standard poultry energy‐evaluation principles (Kong and Adeola 2014; Mahmoud and Ravindran 2025; Hill and Anderson 1958). The nitrogen correction factor of 8.22 kcal/g retained nitrogen was applied for AMEn calculation (Hill and Anderson 1958):

AME(kcal/kg)=(GEintake−GEexcreta)/FI

Nretained=Nintake−Nexcreta

AMEn(kcal/kg)=AME−[8.22×(Nretained/FI)]

Nitrogenretention(%)=[(Nintake−Nexcreta)/Nintake]×100

$$\begin{array}{ccc} & & \text{Apparent}\text{nutrient}\text{digestibility}(\%)=1-[(\text{lignin}\text{diet}/\text{lignin}\text{faeces})\\ & & \;\times \;(\text{nutrient}\text{faeces}/\text{nutrient}\text{diet})]\times 100 \end{array}$$

where GE intake is gross energy intake, GE excreta is gross energy excreted, FI is feed intake, N intake is nitrogen intake, and N excreta is nitrogen excreted. Apparent nutrient digestibility was calculated separately for dry matter and crude protein.

### 2.9 Statistical Analysis

The replicate pen was the experimental unit for diet allocation and statistical analysis. For each analytical module, observations were summarized at the replicate level before analysis. Data were analysed separately for each endpoint using one‐way analysis of variance according to the model *Y_ij_* = *μ* + *T_i_* + *e_ij_*, where *Y_ij_* was the observed response, *μ* was the overall mean, *T_i_* was the fixed effect of diet, and *e_ij_* was the residual error. When the overall *F*‐test was significant, means were separated using Duncan's multiple range test. Statistical significance was declared at *p* < 0.05.

## 3 Results

### 3.1 Energy Utilization and Nitrogen Retention

Diet affected AME, AMEn and nitrogen retention (*p* < 0.001; Table 3). The diet containing 10% FPCW (R2) yielded the highest AME (2948 kcal/kg) and AMEn (2792 kcal/kg). Nitrogen retention in R2 did not differ from that of the higher protein control (RH). In contrast, the 5% and 20% inclusion diets produced the lowest AME and AMEn among the diets containing FPCW. Overall, the response was non‐linear, with the most favourable energy utilization occurring at the moderate inclusion level.

**TABLE 3:** Effects of dietary treatments on apparent metabolizable energy (AME), nitrogen‐corrected apparent metabolizable energy (AMEn) and nitrogen retention in Indonesian super native chickens.

Variable	R0	R1	R2	R3	R4	RH	SEM	*p* value
AME (kcal/kg)	2751^b^	2663^a^	2948^c^	2781^b^	2666^a^	2787^b^	21.83	<0.001
AMEn (kcal/kg)	2599^b^	2525^a^	2792^c^	2634^b^	2519^a^	2635^b^	20.76	<0.001
Nitrogen retention (%)	71.13^b^	62.52^a^	68.73^b^	66.26^b^	65.93^b^	68.30^b^	0.63	<0.001

*Note*: Means within a row with different superscripts differ (*p* < 0.05). *n* = 4 replicates per treatment.

Abbreviation: SEM, standard error of the mean.

### 3.2 Apparent Nutrient Digestibility

Diet also influenced apparent dry matter digestibility and apparent crude‐protein digestibility (*p* < 0.001; Table 4). The 10% inclusion diet again gave the highest response, increasing apparent dry matter digestibility to 63.76% and apparent crude‐protein digestibility to 72.90%. Inclusion above 10% did not provide additional improvement. The higher protein control improved digestibility relative to the low‐protein control, but both digestibility variables remained lower than those observed with 10% FPCW.

**TABLE 4:** Effects of dietary treatments on apparent nutrient digestibility in Indonesian super native chickens.

Variable	R0	R1	R2	R3	R4	RH	SEM	*p* value
Apparent dry matter digestibility (%)	43.00^a^	56.53^c^	63.76^d^	59.19^c^	48.61^b^	47.77^b^	1.58	<0.001
Apparent crude‐protein digestibility (%)	46.50^a^	57.29^c^	72.90^e^	61.14^d^	63.78^d^	50.20^b^	1.86	<0.001

*Note*: Means within a row with different superscripts differ (*p* < 0.05). *n* = 4 replicates per treatment.

Abbreviation: SEM, standard error of the mean.

### 3.3 Selected Ileal Bacterial Counts

Total viable bacteria and *E. coli* counts were not affected by diet (*p* > 0.05; Table 5). In contrast, ileal *S. aureus* counts differed among treatments (*p* = 0.022) and tended to increase as dietary inclusion of FPCW increased, with the highest count recorded in R4. Therefore, the microbiological response was selective and did not indicate a consistent antimicrobial effect under the present conditions.

**TABLE 5:** Effects of dietary treatments on selected ileal bacterial counts in Indonesian super native chickens.

Variable	R0	R1	R2	R3	R4	RH	SEM	*p* value
Total viable bacteria (10^8^ CFU/g)	2.30	2.17	2.12	1.65	1.37	1.60	0.13	0.239
*Escherichia coli* (10^2^ CFU/g)	4.95	4.30	4.25	4.02	3.82	5.10	0.38	0.784
*Staphylococcus aureus* (10^2^ CFU/g)	33.77^a^	37.75^ab^	67.00^bc^	86.00^bc^	95.50^c^	83.00^bc^	7.03	0.022

*Note*: Means within a row with different superscripts differ (*p* < 0.05). Total viable bacteria are expressed as 10^8^ CFU/g, whereas *Escherichia coli* and *Staphylococcus aureus* are expressed as 10^2^ CFU/g. *n* = 4 replicates per treatment.

Abbreviation: SEM, standard error of the mean.

### 3.4 Digestive Enzyme Activities

**FIGURE 1:**
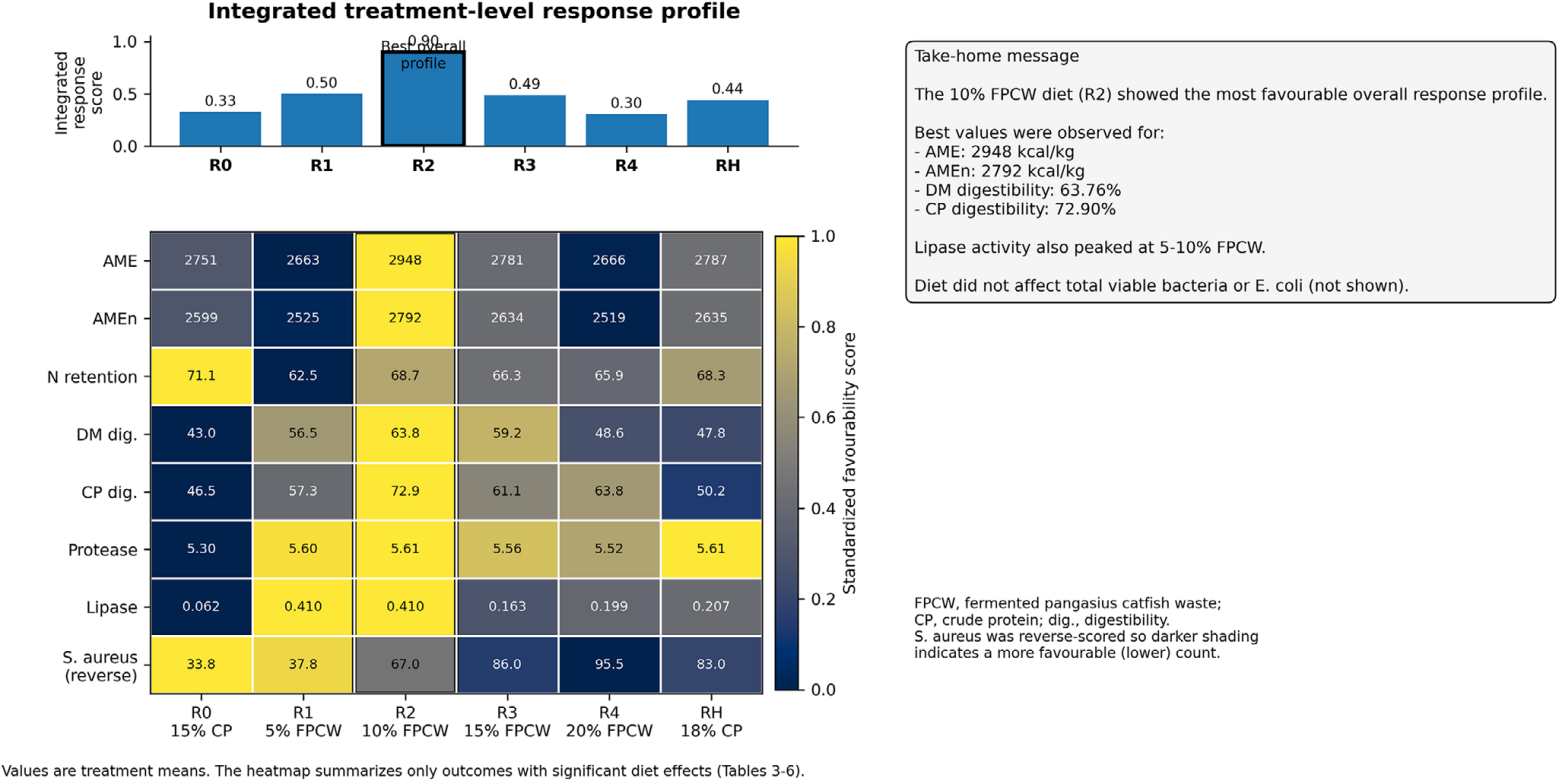
Integrated treatment‐level response profile of Indonesian super native chickens fed low‐protein diets containing fermented pangasius catfish waste (FPCW). The heat map summarizes outcomes with significant diet effects from Tables 3, 4, 5, 6 using min–max standardized treatment means; higher scores indicate more favourable responses, except for *Staphylococcus aureus*, which was reverse‐scored so darker shading indicates lower counts. The upper panel shows the mean integrated response score across significant endpoints. The 10% FPCW diet (R2) showed the most favourable overall response profile. Total viable bacteria and *Escherichia coli* are not shown because diet effects were not significant. AME, apparent metabolizable energy.

Protease and lipase activities were influenced by diet (Table 6). Protease activity was slightly but significantly higher in all diets containing FPCW and in the higher protein control than in the low‐protein control (*p* = 0.020). Lipase activity showed a stronger response (*p* < 0.001), with the highest values observed in R1 and R2. Lipase activity declined at 15% and 20% inclusion, although it remained above the low‐protein control. Taken together, the enzyme data support the view that moderate inclusion improved digestive function more consistently than higher inclusion levels. An integrated visual summary of the treatment‐level response profile is presented in Figure 1.

**TABLE 6:** Effects of dietary treatments on digestive enzyme activities in Indonesian super native chickens.

Variable	R0	R1	R2	R3	R4	RH	SEM	*p* value
Protease (U/mL)	5.30^a^	5.60^b^	5.61^b^	5.56^b^	5.52^b^	5.61^b^	0.03	0.020
Lipase (U/mL)	0.062^a^	0.410^d^	0.410^d^	0.163^ab^	0.199^bc^	0.207^bc^	0.03	<0.001

*Note*: Means within a row with different superscripts differ (*p* < 0.05). *n* = 4 replicates per treatment.

Abbreviation: SEM, standard error of the mean.

## 4 Discussion

The present study identifies 10% FPCW as the most favourable inclusion level within a low‐protein diet for Indonesian super native chickens. At this inclusion level, AME, AMEn and apparent digestibility reached their highest values, whereas nitrogen retention remained comparable with that of the higher protein control. This response pattern is relevant to veterinary production systems because it indicates that a local fish‐processing by‐product can support nutrient use in protein‐reduced diets without simply relying on higher crude‐protein formulation.

The AME and AMEn values of the 10% FPCW diet were 2948 and 2792 kcal/kg, respectively. These values were higher than those of the low‐protein control and were also above the formulated metabolizable energy value of the experimental finisher diets, which was approximately 2750 kcal/kg. For context, commercial broiler nutrition specifications commonly use higher energy densities, with Ross broiler recommendations ranging from approximately 2975 kcal/kg in starter diets to above 3100 kcal/kg in finisher diets (Aviagen 2022). The present AMEn value should therefore be interpreted as favourable for a deliberately low‐protein diet for local chickens, rather than as equivalent to a high‐energy commercial broiler formulation.

The comparison with alternative feed ingredients also supports the practical relevance of the response. Diets containing shrimp waste meal for free‐range chickens have been reported to show AME values ranging from 2527 to 2928 kcal/kg and AMEn values ranging from 2329 to 2774 kcal/kg as inclusion increased (Brito et al. 2020). Energy values of animal by‐products are likewise variable; tilapia processing residue and poultry by‐product meal have shown broad AME and AMEn ranges depending on bird age and ingredient composition (Schneiders et al. 2021). The 10% FPCW diet in the present study falls within this broader range for animal‐derived alternative ingredients, while maintaining a controlled dietary inclusion level and avoiding the decline observed at 15% and 20% inclusion.

The lack of additional benefit above 10% inclusion is equally important. Even when fermentation improves ingredient quality, higher dietary inclusion can increase residual matrix constraints associated with fish by‐products, including ash load, lipid characteristics, mineral imbalance, palatability limitation and source heterogeneity. Similar inclusion‐dependent responses have been reported for aquatic by‐products, fermented shrimp‐derived supplements and other processed alternative proteins, where moderate inclusion generally produces better nutrient use than the highest level tested (Brito et al. 2020; Shabani et al. 2021; Abun et al. 2022; Mirzah et al. 2020; Abun et al. 2021). For practical ration formulation, successful use of fermented by‐products depends on both processing quality and a controlled inclusion rate.

The positive response at 10% inclusion is biologically plausible. Fermentation can increase substrate accessibility before feed manufacture by partially hydrolysing macromolecules, releasing smaller soluble fractions and improving the biological value of protein‐rich by‐products (Dadkhodazadeh et al. 2024; Li and Wang 2021; Sukhikh et al. 2022). The present finding that the 10% FPCW diet outperformed both the unsupplemented low‐protein control and the higher protein control for digestibility suggests that the benefit was not attributable only to crude‐protein concentration. Rather, fermentation likely improved the feeding value of the pangasius waste itself, as also indicated by previous characterization of fermented pangasius waste (Abun et al. 2025a; Abun et al. 2025b).

The enzyme responses partly support the digestibility data. Protease activity increased modestly in FPCW‐containing diets, whereas lipase activity responded most strongly at 5%–10% inclusion and then declined at higher inclusion. This pattern is compatible with the view that moderate dietary inclusion created a more favourable digestive environment than either the unsupplemented low‐protein diet or the higher FPCW diets. Recent poultry studies have similarly shown that nutrient digestibility, enzyme‐related responses and gut traits are sensitive to dietary processing strategy, enzyme supplementation and inclusion level rather than to ingredient identity alone (Songuine et al. 2025; Ogunola et al. 2025; Pourazadi et al. 2024; Salehizadeh et al. 2025; Yi et al. 2024).

In contrast with the clear effects on energy utilization and digestibility, the microbiological response was limited. Fermented ingredients can modify gut ecology, but the magnitude and direction of that effect depend on the substrate, microbial inoculum, processing conditions, host species and selected microbial endpoints (Bromfield et al. 2024; Peng et al. 2022; Zhu et al. 2023; Predescu et al. 2024; Katu et al. 2025). In the present study, total viable bacteria and *E. coli* were unchanged, whereas *S. aureus* counts tended to increase at higher inclusion levels. Accordingly, FPCW should not be presented here as a consistent antimicrobial intervention. Its main value in the present dataset lies in nutrient utilization rather than broad‐spectrum microbial modulation.

The study has limitations. Endpoint data were analysed at the treatment level across separate analytical modules, which means that the strongest inference concerns dietary treatment differences rather than bird‐level associations among variables. Microbiological assessment was restricted to selected culturable indicators and, therefore, does not represent a comprehensive ileal microbiome profile. These limitations narrow mechanistic interpretation, but they do not negate the consistent treatment‐level pattern observed for energy utilization, nutrient digestibility and digestive enzyme activity.

From a practical perspective, the results support 10% FPCW as a realistic local inclusion level for partially replacing conventional protein sources in low‐protein diets for Indonesian super native chickens. This conclusion aligns with the broader poultry literature showing that dietary adaptation to tropical conditions, lower protein feeding strategies and improved processing of alternative feed resources are central to resilient poultry production systems (Kpomasse et al. 2021; Songuine et al. 2025; Ogunola et al. 2025; Pourazadi et al. 2024; Salehizadeh et al. 2025). The present work adds evidence from a locally relevant pangasius‐processing by‐product and provides a cautious, practice‐oriented estimate of its effective inclusion range.

## 5 Conclusions

FPCW can be used as a functional ingredient in low‐protein diets for Indonesian super native chickens. Under the present conditions, 10% inclusion provided the most favourable overall response by improving AME, AMEn, and apparent dry matter and crude‐protein digestibility, while maintaining nitrogen retention comparable with the higher protein control. Responses of selected ileal bacteria were limited and partly unfavourable at higher inclusion levels; therefore, the ingredient should be positioned primarily as a nutrient‐utilization enhancer rather than as a consistent antimicrobial feed additive.

## Author Contributions

*Conceptualization*: Abun Abun. *Methodology*: Abun Abun and Kiki Haetami. *Investigation*: Abun Abun and Rahmad Fany Ramdhan. *Formal analysis*: Abun Abun and Rahmad Fany Ramdhan. *Resources*: Kiki Haetami. *Supervision*: Abun Abun and Kiki Haetami. *Writing – original draft*: Abun Abun. *Writing – review and editing*: Abun Abun, Kiki Haetami and Rahmad Fany Ramdhan.

## Funding

This research was supported by the Ministry of Education, Culture, Research, and Technology (Fiscal Year 2022), with institutional support from Universitas Padjadjaran, Faculty of Animal Husbandry.

## Ethics Statement

All procedures involving animals were reviewed and approved by the Research Ethics Committee of Universitas Padjadjaran, Bandung, Indonesia (Protocol No. 231/UN6.KEP/EC/2023).

## Conflicts of Interest

The authors declare no conflicts of interest.

## Data Availability

The data supporting the findings of this study are available from the corresponding author upon reasonable request.
